# Selectively Etched Halloysite Nanotubes as Performance Booster of Epoxidized Natural Rubber Composites

**DOI:** 10.3390/polym13203536

**Published:** 2021-10-14

**Authors:** Indra Surya, Kamaruddin Waesateh, Abdulhakim Masa, Nabil Hayeemasae

**Affiliations:** 1 Department of Chemical Engineering, Faculty of Engineering, Universitas Sumatera Utara, Medan 20155, Sumatera Utara, Indonesia; isurya@usu.ac.id; 2 Islamic Sciences Demonstration School, Prince of Songkla University, Pattani Campus, Pattani 94000, Thailand; qamarud@hotmail.com; 3 Research Unit of Advanced Elastomeric Materials and Innovations for BCG Economy (AEMI), Faculty of Science and Technology, Prince of Songkla University, Pattani Campus, Pattani 94000, Thailand; abdulhakim.m@psu.ac.th; 4 Rubber Engineering & Technology Program, International College, Prince of Songkla University, Hat Yai, Songkhla 90110, Thailand; 5 Department of Rubber Technology and Polymer Science, Faculty of Science and Technology, Prince of Songkla University, Pattani Campus, Pattani 94000, Thailand

**Keywords:** epoxidized natural rubber, halloysite nanotubes, acid treatment, tensile properties, wide-angle X-ray scattering

## Abstract

Halloysite Nanotubes (HNT) are chemically similar to clay, which makes them incompatible with non-polar rubbers such as natural rubber (NR). Modification of NR into a polar rubber is of interest. In this work, Epoxidized Natural Rubber (ENR) was prepared in order to obtain a composite that could assure filler–matrix compatibility. However, the performance of this composite was still not satisfactory, so an alternative to the basic HNT filler was pursued. The surface area of HNT was further increased by etching with acid; the specific surface increased with treatment time. The FTIR spectra confirmed selective etching on the Al–OH surface of HNT with reduction in peak intensity in the regions 3750–3600 cm^−1^ and 825–725 cm^−1^, indicating decrease in Al–OH structures. The use of acid-treated HNT improved modulus, tensile strength, and tear strength of the filled composites. This was attributed to the filler–matrix interactions of acid-treated HNT with ENR. Further evidence was found from the Payne effect being reduced to 44.2% through acid treatment of the filler. As for the strain-induced crystallization (SIC) in the composites, the stress–strain curves correlated well with the degree of crystallinity observed from synchrotron wide-angle X-ray scattering.

## 1. Introduction

Filler has become a major ingredient in compounding rubber. The main reasons for adding filler to rubber are to improve mechanical properties or thermal stability, and to lower the manufacturing costs [1]. There are plenty of fillers available nowadays in which special attention is given to the incorporation of nanofillers. This is simply because these fillers effectively boost the performance of rubber even at very low contents. This improvement comes from many factors such as the dispersibility, aspect ratio, and orientation of the filler within the matrix [2]. Many types of nanofillers have been used in preparing rubber composites. Here, Halloysite nanotubes (HNT) are the focus. HNT are chemically similar to mineral clay. HNT have been studied in many interesting research works; the applications of HNT are widely known. For example, the use of HNT as drug deliverers, their function in controlled release [3], and other specific applications for ion adsorbents, ceramic materials, especially biocompatible implants, and as templates for the synthesis of rod-like nanoparticles [4]. HNT have also been incorporated into a number of different types of matrices [5,6,7]. The chemical structure of HNT consists mainly of aluminosilicates, which make HNT incompatible with non-polar rubbers such as natural rubber (NR). Thus, searching for a proper technique to address this drawback is of keen interest. Recently, modified rubber as a matrix or compatibilizer has been used in rubber/HNT composites. Surya et al. [5] prepared epoxidized natural rubber as matrix and reinforced it with HNT. Paran et al. [6] also modified the properties of HNT composite by grafting the HNT onto carboxylated nitrile butadiene rubber. From these works, it is clear that higher composite performance is achieved by changing the polarity of the rubber matrix. In this study, turning non-polar NR into a polar characteristic was given special interest. NR was modified into an Epoxidized Natural Rubber (ENR) and was further used as a matrix to assure HNT–rubber compatibility. This solution is well reported in terms of performance [8]. However, it was still unsatisfactory, and the characteristics of HNT themselves could be to blame; in particular, the interfacial filler–matrix adhesion. The specific surface area of HNT controls their interfacial contact with the rubber matrix, so increasing the surface area of HNT could improve the interfacial contact and adhesion.

The surface area of filler is an important factor in its interactions with a polymer matrix. Zhang et al. [9] reported that the BET surface area and pore volume of HNT could be increased by an acid treatment. It was found that the meso-pores were enlarged by a continuous acid treatment, while the micro-porosity was restricted by the crystalline structure. Abdullayev et al. [10] reported that sulfuric acid treatment was an effective method for the controllable enlargement of the lumen diameters of HNT. Sulfuric acid was selected to dissolve alumina sheets out of HNT. Selective etching started with the diffusion of hydrogen ions into HNT pores, followed by interaction between alumina and hydrogen ions and diffusion of the reaction’s product out of the pores. This produced HNT with uniformly enlarged lumen diameters.

Therefore, the aim of this study was to increase the surface area of HNT by using sulfuric acid and further incorporate it into an ENR matrix to test for improvement in the overall properties of the composites. Hypothetically, the interfacial adhesion between the HNT and the ENR would be improved while the rubber–filler interactions would be assured by the polarity of the ENR. The filler modification was expected to improve the compatibility and homogeneity of filler dispersion in the composites, and to thereby enhance the reinforcing efficiency of HNT in filled ENR composites. 

This study also proposes methods for evaluating the reinforcing efficiency in composites using mechanical properties, dynamic properties and strain-induced crystallization (SIC). The last method is considered interesting and not many reports have yet addressed it. It can only be correlated for certain types of rubber, such as NR [11,12,13], because NR has very long polymer chains that crystallize under stretching [14]. This ability to crystallize under strain is due to the high regularity of the molecular structure, as the polymers consist almost entirely of cis-1,4-polyisoprene units [15]. 

Many studies of NR have been carried out combining in situ deformation with X-ray diffraction techniques. Tosaka et al. [16] studied the effects of different crosslink densities on the strain-induced crystallization (SIC) of vulcanized rubber and found that crystallinity developed more quickly in samples with higher crosslink density, but was limited in extent. Toki et al. [17] indicated that the crystallinity increased with strain. They suggested that stretched rubber could fall into three phases, namely a non-oriented amorphous phase, an oriented amorphous phase, and a crystalline phase. SIC of unfilled and filled NR was also assessed by Poompradub et al. [18], who found that the onset strain of SIC decreased after adding filler. The degree of lattice deformation decreased with filler content, especially in carbon black (CB) filled composites. Chenal et al. [19] further explained that different fillers have different characteristics associated with the rubber–filler interactions/reactions. This can either accelerate or slow down SIC depending on chemical crosslink density in the NR matrix. A similar observation was reported in vulcanized NR containing CB particles by Candau et al. [20]. 

Based on the reports above, rubber–filler interactions may speed up crystallization at a certain crosslink density. In this report, we present parallel wide angle X-ray scattering and tensile measurements of ENR composites filled with acid-treated HNT. To date, no report has been published with a detailed investigation concerning the relationship between mechanical and dynamic properties and the SIC of rubber composites. The use of acid-treated HNT reinforced the ENR composites. The results explored in this study give an improved scientific understanding of the role of acid-treated HNT in affecting the overall properties of ENR/HNT composites, and will be useful for the manufacturing of rubber products based on ENR/HNT composites.

## 2. Experimental Details

### 2.1. Materials

High ammonia centrifuged latex (HA) with 60% dry rubber content (DRC) was used to prepare ENR. This latex was centrifuged and supplied by Chalong Latex Industry Co., Ltd., Songkhla, Thailand. The chemicals involved in the synthesis of ENR were Teric N30 as non-ionic surfactant and formic acid and hydrogen peroxide for performic acid reaction, purchased from Sigma Aldrich (Thailand) Co. Ltd., Bangkok, Thailand. The HNT were supplied by Imerys Ceramics Limited, Matauri Bay, New Zealand. The elemental composition of HNT was as follows: SiO_2_ (49.5 wt%), Al_2_O_3_ (35.5 wt%), Fe_2_O_3_ (0.29 wt%), TiO_2_ (0.09 wt%), as well as traces of CaO, MgO, K_2_O, and Na_2_O. Sulfuric acid was supplied by RCI Labscan Ltd., Bangkok, Thailand. Stearic acid was purchased from Imperial Industrial Chemicals (Thailand) Co., Ltd., Bangkok, Thailand. ZnO was supplied by Global Chemical Co., Ltd., Samut Prakan, Thailand. N-cyclohexyl-2-benzothiazole sulfenamide was provided by Flexsys America L.P., Akron, Ohio, USA, and soluble sulfur was bought from Siam Chemical Industry Co., Ltd., Samut Prakan, Thailand.

### 2.2. Preparation of Epoxidized Natural Rubber

The synthesis of ENR was begun by diluting the latex to DRC 15%. Next, 1 phr of non-ionic stabilizer (10% Teric N30) was added while stirring for 30 min at ambient temperature to expel the ammonia dissolved in the HA. The epoxidation was performed using formic acid and hydrogen peroxide at 50 °C in a 10-L glass container at a stirring rate of 30 rpm. The total reaction time was fixed to obtain ENR with 20 mol% epoxide. The epoxide level was characterized as stated in our previous report [8]. The resulting ENR was coagulated with methanol and then washed with water. Finally, it was dried in a vacuum oven at 50 °C prior to use.

### 2.3. Selectively Etching of HNT by Sulfuric Acid

The acid treatment of HNT was conducted according to Zhang et al. [9]. First, a 10 g sample of HNT was added to 100 mL of 3M H_2_SO_4_ solution. The mixture was heated at 70 °C for 2, 4, 6, or 8 h. The acid-treated HNT were then filtered, washed, neutralized for pH, and dried in an oven at 70 °C until reaching a constant weight. The acid-treated HNT was ground in a mortar prior to use in compounding. The surface area of acid-treated HNT was then characterized by BET analysis.

### 2.4. Preparation of ENR/HNT Composites

The recipe for the preparation of ENR/HNT composites is given in Table 1. ENR with 20 mol% epoxide (ENR 20) was compounded with 5 phr of HNT (e.g., untreated or acid-treated HNT depending on the formulation) and the other ingredients except for the curatives (CBS and sulfur) in a Brabender plasticorder (Brabender GmbH & Co. KG, Duisburg, Germany). The fixed amounts of mol% epoxide and HNT were chosen based on the optimum properties obtained from our previous reports [8,21]. The initial mixing temperature was set at 50 °C with a rotor speed of 60 rpm. The compound was then sheeted on a two-roll mill while the curatives were incorporated. Finally, samples of the variously treated composites were tested for curing characteristics.

### 2.5. Measurement of Curing Characteristics

The curing properties of the composites were measured according to ASTM D5289 using a moving die rheometer (Rheoline, Mini MDR Lite, Prescott Instruments Ltd., Tewkesbury, UK). The operating temperature was set at 150 °C. The data in terms of torque, scorch time (ts_2_), and curing time (tc_90_) were recorded as the median values of three repeated tests. The ts_2_ and tc_90_ were used in calculating the curing rate index (CRI) as follows:

(1) $$\mathrm{CRI}=\frac{100}{t_{c90}-t_{s2}}$$

### 2.6. Fourier Transform Infrared-Spectroscopic Analysis (FT-IR)

The changes in functionality of acid-treated HNT and its corresponding composites was confirmed by Fourier transform infrared spectroscopy (FTIR) using FTIR spectroscope model TENSOR27 (Bruker Corporation, Billerica, MA, USA). The spectra were recorded in transmission mode with a 4 cm^−1^ resolution over 4000–550 cm^−1^.

### 2.7. X-ray Diffraction Analysis (XRD)

The XRD analysis of acid-treated HNT and its corresponding composites was carried out using PHILIPS X’Pert MPD (Eindhoven, Netherlands) with CuKα radiation (λ = 0.154 nm) at 40 kV and a current of 30 mA, as well as a Bruker D2 Phaser (Billerica, Massachusetts, USA) with CuKα radiation source (λ = 0.154 nm) and a current of 10 mA. The diffraction patterns were scanned for diffraction angles 2θ at 5–30° with a step size of 0.05° and 3°/min scan speed. The d-spacing of HNT layers in filler particles was estimated using Bragg’s equation.

### 2.8. Measurement of Mechanical Properties and Hardness

Tensile properties were measured according to ASTM D412. The samples were punched with Die C into a dumbbell shape. A universal testing machine (Tinius Olsen, H10KS, Tinius Olsen Ltd., Surrey, UK) was selected to perform the tensile test at a crosshead speed of 500 mm/min. The determinations recorded were the moduli at 100% (M100) and 300% (M300) elongations, tensile strength, and elongation at break. The tear strength of the various composites was tested using the same machine according to ASTM D624. A type C (right angle) test piece was selected for the tests. The last measurement was for the hardness, performed according to ASTM D2240 using a Shore A type manual durometer. The values reported in this section were averages of five repeated tests for each composite.

### 2.9. Determination of Crosslink Density

The crosslink density of the composite was determined by the equilibrium swelling method as described in ASTM D6814. The specimens were cut into a circular shape and weighed before and after immersion in toluene for 72 h. The modified Flory–Rehner equation was implemented for calculating the cross-link density (*υ*) [22]:

(2) $$\nu =\frac{1}{{2M}_{c}}$$

(3) Mc=ρ⋅V0⋅(Vr13−Vr2)ln(1−Vr)+Vr+μ⋅Vr2

where *M_c_* is the number-average molecular weight of the rubber chains between crosslinks, *µ* is the parameter for rubber–toluene interactions (*µ* = 0.42), *ρ* is the bulk density of the specimen, V_0_ is the molar volume of the toluene (*V*_0_ = 106.2 cm^3^/mol), and *V_r_* is the volume fraction in the swollen specimen, defined as follows:

(4) $$V_{r}=\frac{\left(D-\mathrm{FT}\right)\cdot \rho^{-1}}{\left(D-\mathrm{FT}\right)\cdot \rho^{-1}+A_{0}\cdot \rho_{s}^{-1}}$$

where *T* is the weight of the specimen, *D* is the weight of the de-swollen specimen, *F* is the weight fraction of the insoluble parts, *A*_0_ is the weight of the toluene absorbed by the swollen specimen, *ρ* is the density of the specimen, and *ρ_s_* is the density of the toluene (0.886 g/cm^3^). The values were reported as averages of five repeated tests for each composite.

### 2.10. Scanning Electron Microscopy

The freshly fractured surfaces of samples from tensile testing were used to observe the dispersions of untreated and acid-treated HNT in the rubber matrix. The morphology was imaged using a scanning electron microscope (SEM; FEI Quanta FEG 400, Thermo Fisher Scientific, Waltham, MA, USA). Specimens were sputter coated with gold/palladium to eliminate charge buildup during imaging.

### 2.11. Dynamic Properties

The dynamic properties of the composites were implemented in this study to evaluate the rubber–filler interactions through the Payne effect. It was carried out using a Rubber Process Analyzer (RPA), model D-RPA 3000 (MonTech Werkstoffprüfmaschinen GmbH, Buchen, Germany). First, the tested samples were cured at 150 °C based on the t_c90_ as tested using the same RPA. The samples were then cooled down to 60 °C. At this time, at a fixed frequency of 10 Hz, the strain was increased from 0.5 to 90%. This was to determine the storage modulus (*G’*) as function of strain for the composites. The raw *G’* record was further used to study the filler–filler interactions via the so-called Payne effect. The Payne effect was quantified as follows:

Payne effect = *G’_I_* − *G’_f_* (5)

where *G’_i_* and *G’_f_* were the *G’* at 0.5% and 90% strains, respectively. A larger Payne effect indicates weaker rubber–filler interactions. The values were reported in averages of five repeated tests for each composite.

### 2.12. Wide-Angle X-ray Scattering

The SIC of the composites was correlated with their stress–strain curves. SIC and other related results were obtained via a synchrotron wide-angle X-ray scattering (WAXS) analysis. The experiment was carried out using Beamline 1.3 W at the Siam Photon Laboratory, Synchrotron Light Research Institute (SLRI), Nakhon Ratchasima, Thailand. The distance between sample and detector was 115.34 mm, measured using a wavelength of 0.138 nm. A CCD detector (Rayonix, SX165, Rayonix, L.L.C., Evanston, IL, USA) with a diameter of 165 mm was used to capture the WAXS profile. The scattering angle was calibrated using 4-Bromobenzoic acid as the standard material.

Prior to testing, a Die C type dumbbell specimen was placed in the grips of a stretching apparatus. The sample was stretched at a crosshead speed of 50 mm/min to a given strain and was then relaxed in the deformed state for 30 s. WAXS was recorded and stretching then continued to the next predetermined strain, repeating until the characterization was complete. The degree of crystallinity (*X_c_*) was calculated based on data obtained from the WAXS profiles as follows:

(6) $$\mathrm{Degree}\;\mathrm{of}\;\mathrm{crystallinity}\;(X_{c})=\left(\frac{A_{c}}{A_{c}+A_{a}}\right)\times 100$$

where *A_c_* and *A_a_* are the areas under the crystalline peak of interest and the amorphous halo, respectively.

The orientation parameter (OP) was determined from the Hermann equation, as follows:

(7) $$\mathrm{OP}=\frac{{3[\cos}^{2}\phi ]-1}{2}$$

where *φ* is the azimuthal angle related to the direction of strain. The mean value of cos^2^ *φ* is calculated as follows:

(8) $${[\cos}^{2}\phi ]=\frac{\int_{0}^{\pi }I_{c}(\phi )\cdot \cos^{2}\phi \cdot \sin\phi \cdot d\phi }{\int_{0}^{\pi }I_{c}(\phi )\cdot \sin\phi \cdot d\phi }$$

where *I_c_* (*φ*) is the scattering intensity of the crystal at *φ*. *I_c_* (*φ*) is normalized by subtracting the minimum scattering intensity of the amorphous component of the original WAXS intensity [23,24]. The data reported in this section were the median values of three repeated tests.

## 3. Results and Discussion

### 3.1. BET Surface Area of HNT

**Figure 1:**
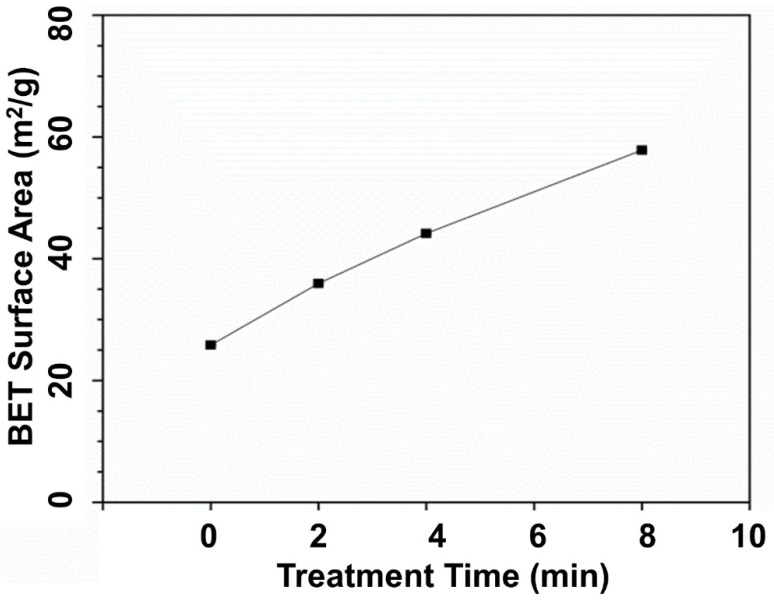
Specific surface area of HNT by acid treatment time.

**Figure 2:**
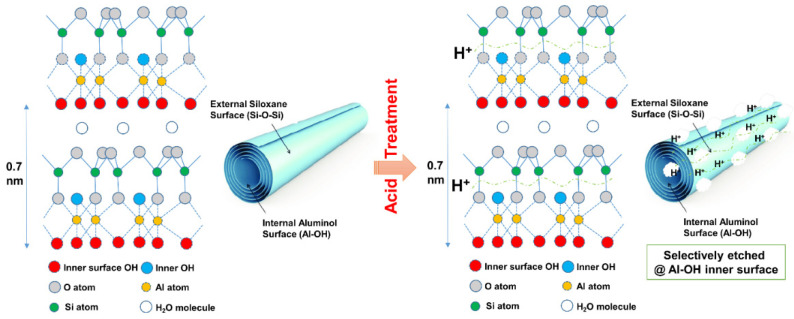
Possible etching mechanism of HNT by sulfuric acid.

The main reason to treat the HNT with acid was to increase their specific surface area in order to gain improved contact with the rubber matrix. To assess this expectation, the surface area of acid-treated HNT was measured via the BET technique. The BET surface areas of raw HNT and acid-treated HNT are presented in Figure 1. The BET surface area of HNT increased from 25.83 to 57.83 m^2^/g with acid treatment time. The larger specific surface area found was attributed to etching by H_2_SO_4_, and specifically to the leaching of Al^3+^ ions from the octahedral layer due to hydrolysis under acidic conditions. The reaction between kaolinite and sulfuric acid, according to Makó et al. [25], can be expressed as follows: Al_2_O_3_·2SiO_2_·2H_2_O + 3H_2_SO_4_ → Al_2_(SO_4_)_3_ + 2SiO_2_ + 5H_2_O. The etching of HNT surfaces by sulfuric acid also reduced hydroxyl groups attached on the Al–OH inner surfaces of HNT, due to the penetration of sulfuric acid into the inner layers of HNT. The possible etching mechanism of sulfuric acid on HNT is illustrated in Figure 2. This mechanism is further correlated with the FTIR results in the following section.

### 3.2. FT-IR Analysis

**Figure 3:**
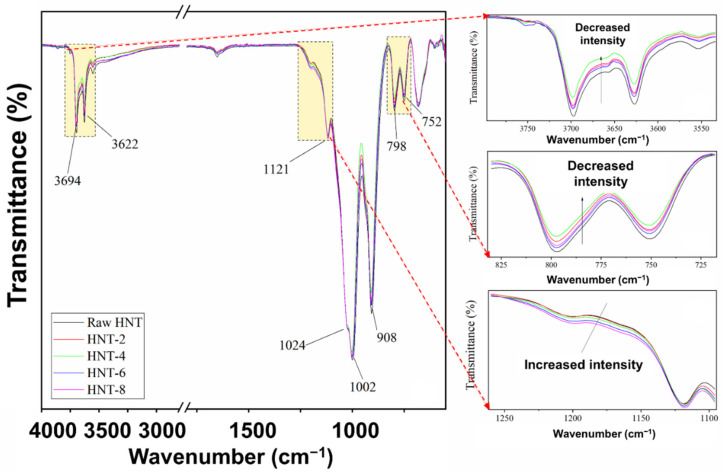
Infrared spectra of HNT powder after various durations of acid treatment.

To confirm the structure of HNT before and after acid treatment, FTIR spectra of raw HNT and acid-treated HNT were captured and are shown in Figure 3. In the O–H stretching region, the untreated HNT and acid-treated HNT showed bands at 3694 cm^−1^ and 3622 cm^−1^, which correspond to inner surface and outer surface hydroxyl groups stretching, respectively. The acid treatments applied to the HNT did not show significant variation in FTIR patterns. In the fingerprint region, the HNT showed a series of bands with peaks at wavenumbers 908 cm^−1^, 798 cm^−1^ and 752 cm^−1^ that can be assigned to the Al–Al–OH, Al–Mg–OH and Si–O–Al vibrations in the HNT sheet. The strong bands in the 1120–1000 cm^−1^ region were due to Si–O stretching, which was observed for both untreated and acid-treated HNT [26]. The reductions in peak intensity in the regions 3750–3600 cm^−1^ and 825–725 cm^−1^ were attributed to decreased numbers of Al–OH structures, while the increased absorption intensity in the region 1250–1100 cm^−1^ was associated with silicon-rich nanoparticles [27] formed after the destruction of HNT structures by acid treatment. Such richness in Si–O possibly increased the interaction with ENR, which will be discussed further in the following section.

**Figure 4:**
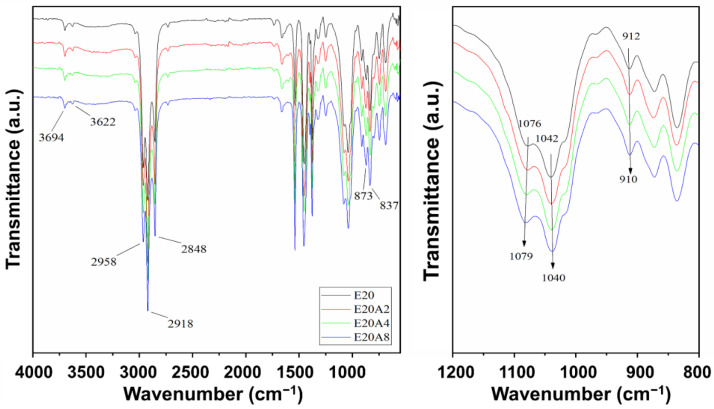
Infrared spectra of ENR/HNT composites filled with untreated and acid-treated HNT.

Figure 4 displays the FTIR spectra in the wavenumber range 4000–550 cm^−1^ for ENR/HNT composites filled with untreated and acid-treated HNT. The important absorption peaks indicating stretching vibrations of C=C bonds, bending vibrations of CH_2_ and CH_3_ groups, and out of plane deformation of =C-H groups were found at 1662 cm^−1^, 1448 cm^−1^ and 1375 cm^−1^, and 837 cm^−1^ respectively. The absorption peaks at 873 cm^−1^ and 1250 cm^−1^ indicated the epoxide rings in ENR. The hydroxyl group formed from the ring-opening of ENR is shown in the broad peak region at approximately 3400 cm^–1^ [28,29]. The peaks at approximately 1100–1020 cm^−1^ and 912 cm^−1^ are assigned to stretching vibrations of Si–O bonds and Al–OH, respectively. The shifting of peaks from 1076 cm^−1^ to 1079 cm^−1^ (see the enlarged image) indicates some interactions caused by hydrogen bonding between ENR and hydroxyl groups on the edge of HNT.

### 3.3. X-ray Diffraction Analysis

**Figure 5:**
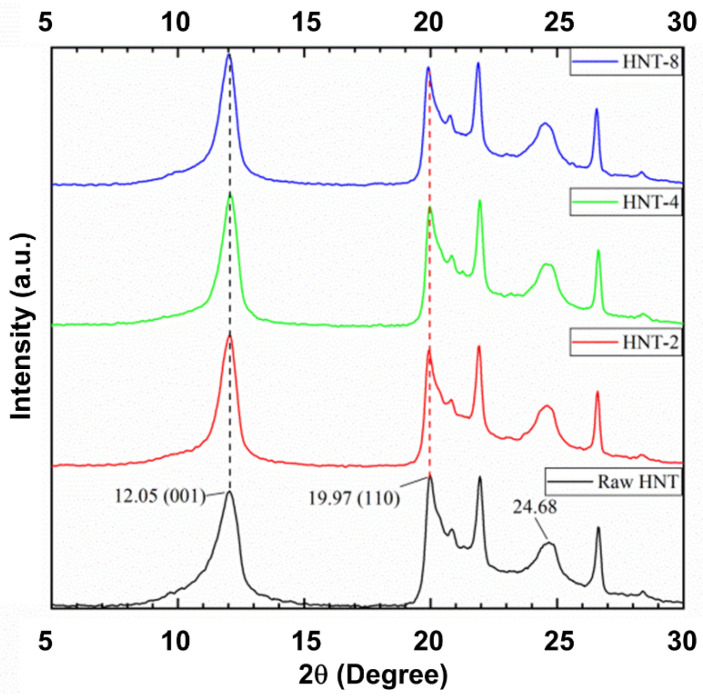
XRD scattering patterns of HNT after various durations of acid treatment.

Figure 5 shows the XRD profiles of untreated and acid-treated HNT. The untreated HNT showed reflections at 2θ of 12.05° and 24.68°, which correspond to a d_001_ basal spacing of 7.33 Ǻ [26]. Generally, the XRD pattern can change after acid treatment, as verified in the study by Makó et al. [21]. As the structure of HNT was destroyed, there was a change in the crystal structure of HNT. However, this change was small. The minor change might be ascribed to the use of a low acid concentration [30]. Moreover, the original HNT phase (001) became thinner when compared to untreated HNT, as also reported by Panda et al. [26], since low concentration acid was applied to kaolinite. The narrowing of the peak may be related to the increase in crystallite size of the HNT structure.

**Figure 6:**
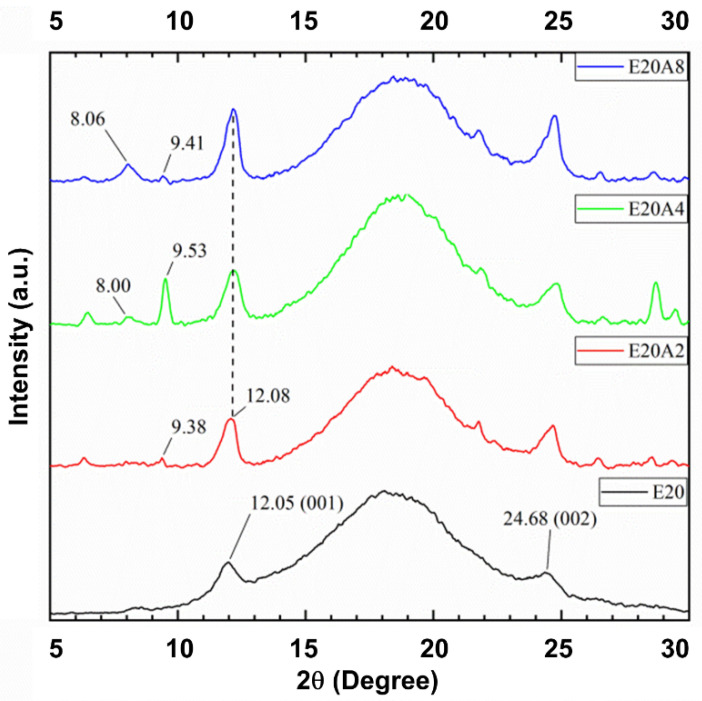
XRD scattering patterns of ENR/HNT composites filled with untreated and acid-treated HNT (un-deformed specimen).

Figure 6 shows XRD patterns of the ENR/HNT composites filled with untreated and acid-treated HNT. For the untreated-HNT filled composite, general characteristics of peaks were observed at 2θ of 12.05° (d is 7.33 Ǻ).This is simply due to the 001 plane in HNT structure. Upon using acid-treated HNT, there was a shift of this peak from 12.05° (d is 7.33 Ǻ) to 8.00° (d is 11.04 Ǻ). The shift of 2θ could be associated with a change in HNT basal spacing caused by octahedral structure etching, assuming that the HNT coil was enlarged, which led to a possible intercalation of rubber and other ingredients [31,32,33]. Once the intercalation occurred, there were possible interactions between ENR and HNT in the system.

### 3.4. Curing Characteristics

**Figure 7:**
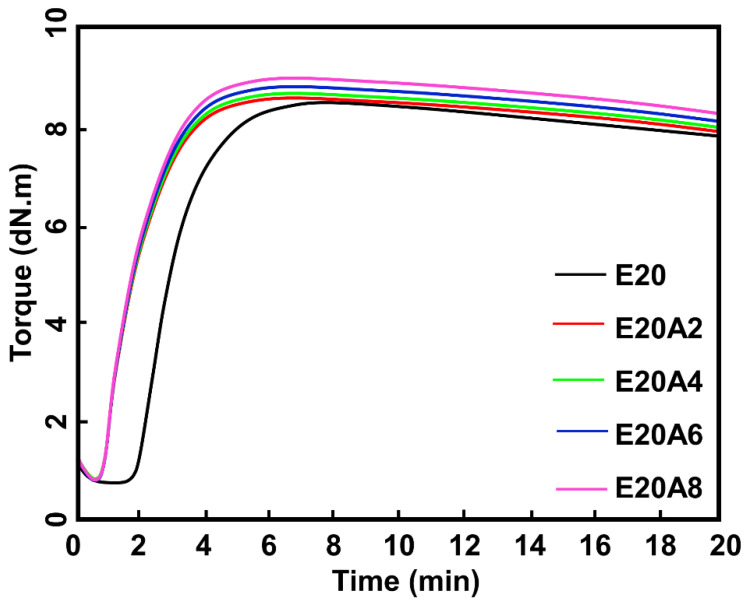
Rheometric curves of ENR/HNT composites filled with untreated and acid-treated HNT (un-deformed specimen).

Rheometric curves of ENR/HNT composites filled with untreated and acid-treated HNT are shown in Figure 7, and the raw outputs obtained from those curves are summarized in Table 2. The scorch time (t_s2_) and cure time (t_c90_) decreased considerably, which reflected an increase in CRI. According to Zhang et al. [9], treatment of HNT with acid increases the amount of silanol groups on the outer layer of HNT. The epoxide groups available on the ENR backbone can interact more easily with silanol groups. The accelerator, which is most likely to be adsorbed on the HNT surfaces, acted efficiently during the vulcanization process. The M_L_ of the composites filled with untreated and acid-treated HNT reduced with treatment time. Since the M_L_ correlates with the viscosity of the compound, it can be said that the destruction of HNT by acid-etching may have affected the viscosity of the compounds. However, M_H_ and M_H_-M_L_ tended to increase with acid treatment time. In composite materials, the M_H_ usually indicates the stiffness of cured compounds and reflects their crosslinking and/or interactions. This is considered a preliminary indicator of interactions between ENR and acid-treated HNT, which were already confirmed by the previous FTIR and XRD results.

### 3.5. Dynamic Properties

**Figure 8:**
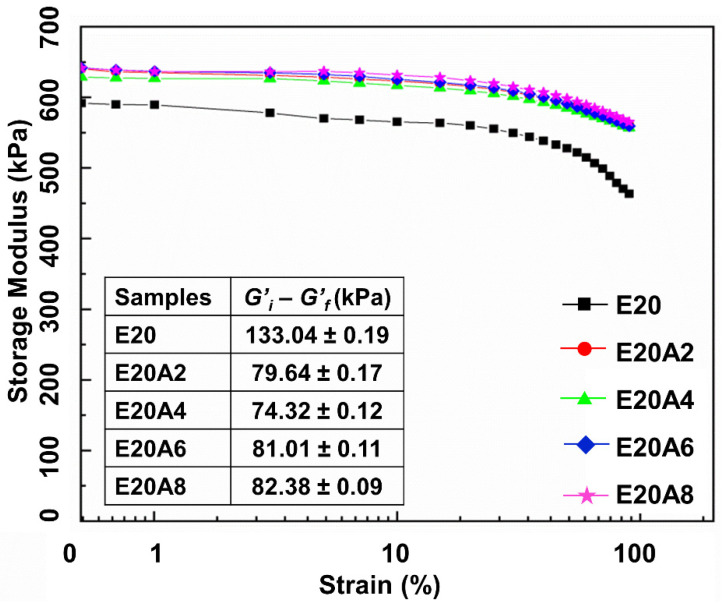
Strain dependence of storage modulus and delta G’ for ENR/HNT composites filled with untreated and acid-treated HNT.

**Figure 9:**
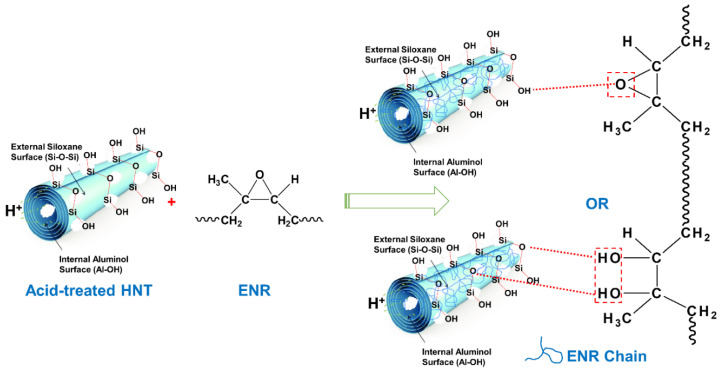
Possible interactions of ENR with acid-treated HNT filler.

One approach to assessing possible interactions in a composite is by use of the dynamic mechanical properties. In this study, storage modulus and the Payne effect of the ENR/HNT composites filled with untreated or acid-treated HNT filler were analyzed to assess the rubber–filler interactions. The results are presented in Figure 8. The storage modulus (*G’*) of the composites was constant in the low strain region but slightly decreased with strains larger than 50%. This is common for a viscoelastic material and is due to the molecular stability of rubber. It is noticeable that the *G’* increased with acid treatment time, indicating interactions between acid-treated HNT and ENR that resulted in a stronger elastic response. There were two factors determining the increase in *G’*: better interfacial adhesion of HNT facilitated by acid treatment, together with improved interactions between polarity matching HNT and ENR. The interactions between ENR and acid-treated HNT are made clear by the mechanisms illustrated in Figure 9. The interactions were through hydrogen bonds between epoxide groups and/or ring openings, and the silanol and siloxane groups of HNT, respectively. The interactions between acid-treated HNT and ENR were clearly supported by the previous FTIR and XRD observations. The shifting of 2θ due to a change in HNT basal spacing together with the shifting of peaks from 1076 cm^−1^ to 1079 cm^−1^ significantly indicated certain interactions between ENR and hydroxyl groups on the edge of HNT. Furthermore, the Payne effect relates to the rubber–filler interactions in the composite, and its measure here was the difference in *G’* between low and high strain [34]. Higher values of delta *G’* indicates higher Payne effect which later reflects to lesser rubber–filler interactions. It was found that the Payne effect decreased with treatment time, where the value of the Payne effect was found to be reduced to 44.2% after treatment with sulfuric acid for 4 h. This might be due to stable rubber–filler interactions involving acid-treated HNT filler in the ENR matrix. 

### 3.6. Mechanical Properties and Corresponding Morphologies

**Figure 10:**
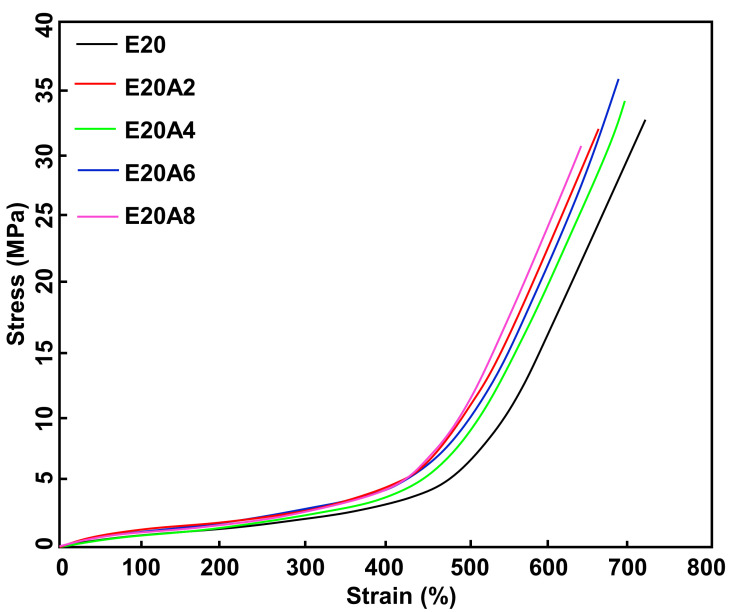
Stress-strain relationships of ENR/HNT composites filled with untreated and acid-treated HNT.

Figure 10 shows the stress–strain curves of the ENR composites filled with untreated and acid-treated HNT. The stress–strain curves show the SIC. A higher stress response was found for the composites filled with acid-treated HNT, suggesting that the samples became stronger when using acid-treated HNT. Improved compatibility of ENR with acid-treated HNT is responsible for these findings. Further, the area underneath the stress–strain curve was examined to confirm the compatibility of the rubber and the filler. This indicates the toughness of a material [35]. A larger area underneath the curve corresponds to greater toughness. The acid-treated HNT composites showed a greater area underneath the stress–strain curve than the untreated counterpart, and therefore greater toughness. The curves shown are further discussed regarding crystallization behavior.

**Figure 11:**
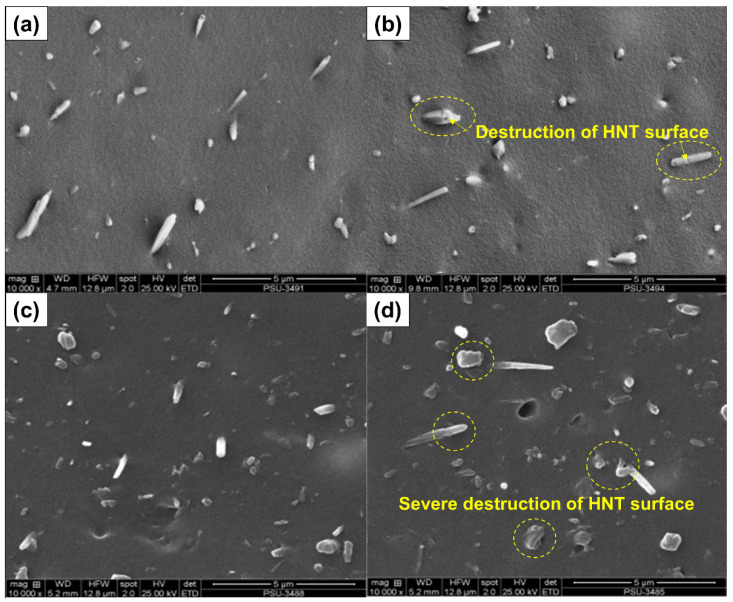
SEM photographs at 10,000× magnification of ENR/HNT composites filled with untreated and acid-treated HNT: E20 (**a**), E20A4 (**b**), E20A6 (**c**), and E20A8 (**d**).

Table 3 summarizes the raw data obtained from tensile, tear and hardness measurements. The tensile and tear strengths changed with the modification of HNT over the duration of acid treatment. The tensile strength with untreated HNT was 33.67 MPa and increased to 35.45 MPa on treating HNT for 4 h. The tear strength of the reference sample increased from 38.29 N/mm to 38.38, 39.60, 37.51, and 35.96 N/mm at 2, 4, 6 and 8 h respectively. Acid treatment of HNT evidently improved interfacial adhesion via the increased specific surface area of HNT. Evidence of such boosting has already been shown in the previous sections (refer to the Payne effect). The decrease in tensile and tear strengths with treatment times over 4 h may be due to some damage to the HNT from the etching process, as seen in SEM images. The SEM images (see Figure 11a–d) show that there was little damage to the HNT surface at 4 h of treatment, while the HNT was well distributed throughout the ENR matrix. However, with a longer acid treatment, severe destruction of HNT was seen (Figure 11c,d), together with some agglomeration of HNT filler into the ENR matrix. These observations match the reduced tensile strength of the composites observed with prolonged acid etching treatments.

The significant change in the rubber–filler interactions of ENR and HNT can be also verified from the stresses at 100% (M100) and 300% (M300) strains (see Table 3). It can be seen that the M100 and M300 increased with acid treatment time. As HNT with longer treatment times was introduced to the rubber, stronger interactions occurred, resulting in harder and stiffer composites. This finding is clearer when examining the M300. The results match well the reduction in elongation at break of the composites, which was due to lower flexibility of molecular chains contributed by the filler–matrix interactions. The observed modulus trend well matches the hardness observations where similar discussion can be implemented.

### 3.7. Wide-Angle X-ray Scattering

**Figure 12:**
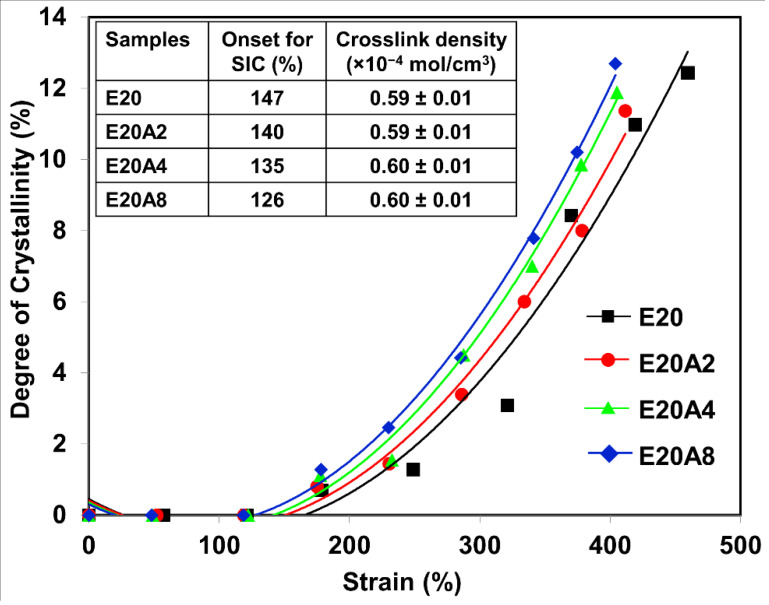
Strain dependence of the degree of crystallinity in ENR/HNT composites filled with untreated and acid-treated HNT.

In the section on mechanical properties, the stress–strain behavior of the composites was associated with strain induced crystallization (SIC). Since the nominal strain rates for tensile measurement and SIC study are not similar (e.g., 0.42 s^−1^ and 0.042 s^−1^ for tensile test and WAXS, respectively), the correlation was made for stress versus crystallinity only. Previously, it was clear that the treatment of HNT with acid influenced the mechanical properties. The main factor was definitely the improved compatibility between the ENR matrix and the acid-treated HNT filler. The degree of crystallinity (*X_c_*) versus strain deformation is shown in Figure 12. Crystallinity was estimated from the areas in diffraction patterns for 200 and 120 plane reflections [36,37]. The *X_c_* increased with strain due to molecular chain orientation, as expected. The onset strain for SIC was determined from intercept of a regression line for *X_c_* as a function of strain (see the data embedded in Figure 12). The onset of SIC for acid-treated HNT filler was observed to decrease as acid treatment was prolonged. The interaction that takes place in the presence of acid-treated HNT can help pull the surrounding molecular chains and speeds up the crystallization process. 

When considering the *X_c_* and the stress propagation obtained via tensile measurement (see Figure 10), it is clear that the *X_c_* corresponded well with stress, and the trend of the curves was also similar over treatment time. From the stress–strain curves, it is obvious that the stress began to increase as treatment time increased. This is attributed to interfacial contacts, as discussed earlier. An earlier onset of SIC is usual for filled composites. Poompradu et al. [19] reported that the lateral crystallite size decreased, but the orientational fluctuation increased upon inclusion of filler. The lattice of the SIC changed almost linearly with the nominal stress. In addition, the degree of lattice deformation decreased with the filler content, especially in the CB-filled system. In addition to this, onset of SIC was dependent on the filler characteristics. Ozbas et al. [38] compared the SIC of graphene and CB-filled composites. They found that the onset of SIC occurred at significantly lower strain for graphene-filled NR samples compared with CB-filled NR, even at low loadings. Chenal et al. [20] further explained that the onset of SIC is ruled by the strain amplification induced by the filler. Moreover, additional interactions in the rubber network are responsible for either accelerating or slowing down the crystallization rate, depending on rubber matrix chemical crosslink density. Ozbas et al. [38], together with the report of Candau et al. [39], further emphasized that rubber–filler interactions may hasten SIC at low crosslink density. This is because high crosslinking may interfere with the chain orientation and reduce SIC. Therefore, the crosslink density of this composite was also reported (see the data embedded in Figure 12). It can be seen that the crosslink density observed was fairly constant across the cases. This is a good indication that network chain density was not involved in the development of SIC, regardless of the treatment time. As a consequence, the change in SIC is attributed to rubber–filler interactions.

**Figure 13:**
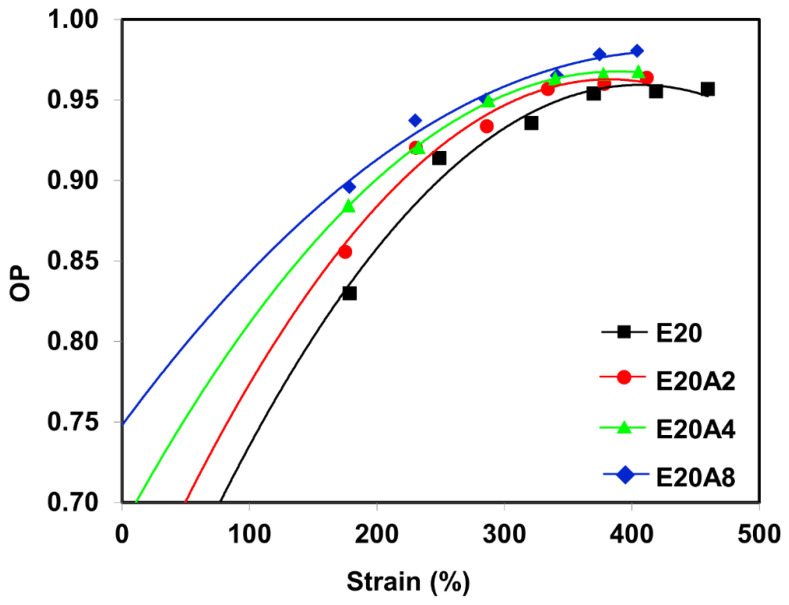
Strain dependence of the orientation parameter for ENR/HNT composites filled with untreated and acid-treated HNT.

The orientation parameter (OP) indirectly indicates the molecular chain orientation and alignment and can be estimated from the Herman equation [23,24]. The OP for the composites is shown in Figure 13. Completely oriented molecular chains would have an OP of one [40]. Here, the OP for the composites was smaller at low strain and grew with increasing strain, confirming that stretching oriented the molecular chains. The composites filled with acid-treated HNT showed higher OP values at low strain, indicating that the acid modification of HNT increased rubber–filler interactions, and accordingly, stronger molecular chain orientation was found for composites filled with acid-treated HNT.

**Figure 14:**
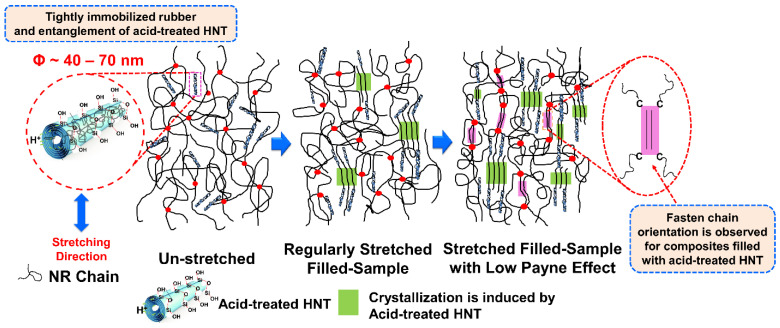
Schematic model representing the crystallization development of ENR/HNT composites filled with acid-treated HNT.

Based on the above findings, the correlation between the SIC and corresponding interactions between ENR and untreated or acid-treated HNT are represented in the schematic model of Figure 14. Referring to the scheme, nothing happened when the sample was not stretched; the ENR matrix may have been in contact with the HNT due to the interfacial interactions resulting from the unique characteristics of the HNT and the polar sites of ENR. When strain was applied to the sample, crystallization of the ENR was induced (SIC), and the crystallinity increased in association with the orientation of the HNT. HNT were oriented and aligned to the stretching direction. This always happened regardless of whether untreated or acid-treated HNT was used. This is usual for filled composites, as has been reported elsewhere [20,38,39]. However, it is interesting to note that the crystallinity of the ENR matrix increased steadily due to the collaborative crystallization of ENR and acid-treated HNT. Higher rubber–filler interactions, as indicated by a lower Payne effect, were responsible for this change. The presence of the acid-treated HNT played an important role in pulling the surrounding molecular chains. Thus, a significant increase in crystallization was observed at larger strains, and this is in agreement with the results from stress–strain behavior and WAXS profiles.

## 4. Conclusions

Selectively etched HNT were successfully prepared using sulfuric acid. This was confirmed by FTIR and XRD. At first, the specific surface area of HNT was found to increase with treatment time. This was seen from BET surface analysis, increasing from 25.83 m^2^/g to 57.83 m^2^/g with increasing acid treatment time. The FTIR showed a decrease in Al–OH structures, as indicated by reduced peak intensities in the regions 3750–3600 cm^−1^ and 825–725 cm^−1^. Upon using acid-treated HNT, there was a shift of the XRD peak from 12.05° (d-spacing is 7.33 Ǻ) to 8.00° (d-spacing is 11.04 Ǻ), indicating that the HNT coil was enlarged, possibly enabling intercalation by rubber and other ingredients. The modification of HNT with acid resulted in accelerated t_s2_, and t_c90_, M_H_ and M_H_–M_L_ were influenced by the improved interactions between the ENR matrix and the acid-treated HNT filler. Increased tensile strength and tear strength were also observed due to the improved filler–matrix interfacial adhesion with acid-treated HNT in the rubber matrix. The percentage increments of tensile and tear strengths for the composites filled with untreated HNT and acid-treated HNT (4 h) were 5.3% and 3.4% respectively. This was further confirmed by the dynamic properties of the composites, as the value of Payne effect was clearly reduced to 44.2% when using HNT treated for 4 h. The SIC in the composites exhibited a clear change, as the strain upturn occurred at a lower strain during stretching, indicating faster crystallization caused by better interfacial interactions within the composites. The *X_c_* was directly observed with XRD during stretching, and corresponded well with the tensile moduli of the composites. Based on the observations overall, it can be concluded that a treatment of HNT with sulfuric acid for 4 h is highly advantageous for preparing composites with an ENR matrix, improving filler–matrix compatibility and strength of the composite vulcanizates. In addition, treatment of HNT by sulfuric acid can be the solution of choice for boosting ENR–HNT interactions. It can promote these improvements without requiring the use of complicated and costly silane coupling agent systems.

## Figures and Tables

**Table 1:** Formulation of ENR composites filled with untreated and acid-treated HNT.

Raw Material	Amount (phr)
ENR 20	100.0
Stearic acid	1.0
Zinc oxide	5.0
HNT *	5.0
CBS	2.0
Sulfur	2.0

Remark: * HNT was acid-treated with various treatment times.

**Table 2:** Scorch time (ts_2_), cure time (tc_90_), minimum torque (M_L_), maximum torque (M_H_), delta torque (M_H_–M_L_), and CRI for the ENR/HNT composites produced with untreated or acid-treated HNT.

Sample	Ts_2_ (min)	Tc_90_ (min)	M_L_ (dN·m)	M_H_ (dN·m)	M_H_–M_L_ (dN·m)	CRI (min^−1^)
E20	2.29	4.75	0.76	8.40	7.64	40.65
E20A2	1.11	3.15	0.81	8.53	7.72	49.02
E20A4	1.10	3.05	0.77	8.58	7.81	51.28
E20A6	1.07	3.01	0.75	8.63	7.88	51.55
E20A8	1.05	2.92	0.74	8.88	8.14	53.48

**Table 3:** Modulus at 100% (M100), 300% (M300), tensile strength (TS), elongation at break (EB), tear strength (Ts), and hardness of ENR/HNT composites filled with untreated and acid-treated HNT.

Sample	M100 (MPa)	M300 (MPa)	TS (MPa)	EB (%)	Ts (N/mm)	Hardness (Shore A)
E20	0.86 ± 0.03	2.25 ± 0.03	33.67 ± 1.61	717 ± 10	38.29 ± 0.94	39.3 ± 0.3
E20A2	0.89 ± 0.02	2.50 ± 0.07	34.84 ± 0.90	676 ± 30	38.38 ± 0.90	41.4 ± 0.5
E20A4	0.92 ± 0.02	2.59 ± 0.08	35.45 ± 0.90	658 ± 22	39.60 ± 0.60	42.1 ± 0.2
E20A6	0.93 ± 0.02	2.62 ± 0.12	32.15 ± 0.40	655 ± 25	37.51 ± 1.21	42.8 ± 0.8
E20A8	0.95 ± 0.02	2.72 ± 0.05	31.00 ± 0.37	652 ± 26	35.96 ± 1.08	43.2 ± 0.4

## Data Availability

The data presented in this study are available on request from the corresponding author.
